# Evidence for the existence of dopamine D2R and Sigma 1 allosteric receptor-receptor interaction in the rat brain: role in brain plasticity and cocaine action

**DOI:** 10.1186/2193-1801-4-S1-P37

**Published:** 2015-06-12

**Authors:** Luca Pinton, Dasiel O Borroto-Escuela, Manuel Narváez, Julia Oflijan, Luigi F Agnati, Kjell Fuxe

**Affiliations:** Department of Neuroscience, Karolinska Institutet, Sweden; Department of Physiology, School of Medicine, University of Málaga, Spain

**Keywords:** Dopamine D2 receptor, Sigma 1 receptor, heteroreceptor complexes

Drug addiction is regarded as one of the most important neuropsychiatric diseases afflicting our society today. A prototypic drug of abuse is cocaine which directly acts on the brain reward system. In this work we present evidence on the existence of dopamine D2R- Sigma1R heteroreceptor complexes which may play a role in the etiology of cocaine addiction. By means of BRET D2R-Sigma1R heteromers were demonstrated in HEK293 cells after receptor cotransfection. The existence of D2R-Sigma1R heteroreceptor complexes was demonstrated also in discrete regions of the ventral and dorsal striatum with in situ proximity ligation assay. Through saturation binding assay it was clearly demonstrated that in membrane preparations of HEK293 cells co-expressing D2R-Sigma1R, cocaine (1nM) significantly increased the D2R Bmax values (998±40 fmol/mg protein) over D2R alone cells (664±37 fmol/mg protein). This effect was counteracted by the Sigma1R selective antagonist PD144418 (Bmax value: 728±39 fmol/mg protein). Furthermore, CREB reporter luc-gene assay indicated that the presence of D2R-Sigma1R significantly reduced the potency of the D2R like agonist quinpirole to inhibit the forskolin induced increase of the CREB signal. In contrast, the presence of a low concentration of cocaine (100nM) was found to markedly increase the quinpirole potency to inhibit the forskolin induced increase of the CREB signal in the D2R-Sigma1R cells. These dynamic changes in D2R-Sigma1R signalling produced by cocaine maybe explained by synergistic allosteric receptor-receptor interactions in the D2R-Sigma1R heteroreceptor complexes at the plasma membrane level. An antagonistic allosteric receptor-receptor interaction between the dopamine D2R and the Sigma1R in absence of cocaine instead of can explain the reduced potency of quinpirole. These dual conformational changes in the D2R-Sigma1R heteroreceptor complexes could be associated with the redistribution of both protomers from the intracellular compartment to the plasma membrane as indicated by means of confocal analysis of agonist induced D2RSigma1R trafficking and internalization. Overall, the dynamic of D2R-Sigma1R heteroreceptor complexes may represent a mechanism that shapes neuronal and addictive responses to cocaine.

